# The government of masks in sentinel territories against Covid-19: Dakar and Seine-Saint-Denis

**DOI:** 10.1186/s12889-023-15968-2

**Published:** 2023-07-05

**Authors:** Frédéric Keck, Enguerran Macia

**Affiliations:** 1 French National Centre for Scientific Research Laboratoire d’anthropologie sociale (CNRS, UMR 7130), 52 rue Cardinal Lemoine, 75005 Paris, France; 2 Santé, Sociétés (CNRS, UMI3189), Faculté de Médecine, French National Centre for Scientific Research Environnement, Université Cheikh Anta Diop de Dakar (UCAD), BP 5005, Dakar Fann, Sénégal

**Keywords:** Face mask, Covid 19, State, Public space

## Abstract

The pandemic of Covid-19 has led to reluctance or resistance to wear a mask in countries that made it compulsory. The acceptance to wear a mask against respiratory diseases depends on conceptions of scientific authority and of the personality in the public space. It has material and symbolic dimensions that can be covered under the term “government of masks”. We have questioned populations on these two aspects in territories we call sentinel because they are more exposed than others to emerging infectious diseases: Dakar (Senegal) and Seine-Saint-Denis (France). In France, school students have asked 250 people in public places on their perception of masks following a questionnaire, while in Senegal a team of master students went in 606 people’s houses to ask questions following the same questionnaire. Despite these methodological differences, our results show that the perception of the State in these territories influences the uses of masks against Covid-19 at symbolic and material levels. While in Seine-Saint-Denis, the State provides more masks than in Dakar, the trust in the efficacy of mask wearing is lower. From a symbolic point of view, the mask is for many people an intrusion of the State into the private sphere, which hinders physical contact between family members. On the contrary, from the material point of view, the mask is a need that manifests the presence of the welfare State to watch over the health of the population. This comparative study suggests that a State that is at the same time coercive and protective produces a lower level of adherence to mask-wearing recommendations than a State where religious authorities are included in the prescription and where individuals contribute to making masks.

## Introduction

The pandemic caused by the coronavirus SARS-Cov2, called Covid-19 by the World Health Organization, was identified in China in December 2019 and spread worldwide, causing the death of more than 6 million people. In the absence of a vaccine in the early stages of the pandemic, social distancing measures and the wearing of surgical masks became necessary to resume social life after the more severe measures of containment, quarantine or curfew, put in place by governments. The surgical mask is simple to manufacture, inexpensive, and its effectiveness in containing viral particles has been demonstrated in several studies [1–3]. However, the reluctance, or even resistance, that the recommendation or obligation to wear a mask as a health measure may have aroused, makes it necessary to analyze the anthropological and sociological reasons that lead people to adhere or not to these public health campaigns [4, 5].

The objective of this article is to analyze the reasonings associated with the wearing of masks in territories that we will call “sentinel”, i.e. in which the populations are particularly vulnerable to emerging diseases because of the density and diversity of their population, but also because of the precariousness of their living conditions. Based on original data collected before the dissemination of vaccines in Dakar, the economic and political capital of Senegal, and in the department of Seine-Saint-Denis, a poor suburb close to the French capital, we have sought to understand the relationship between the State and individuals through the mask, considered as a material and symbolic interface. This analysis could, in fine, be used to better communicate with the populations of sentinel territories in the event of a future epidemic transmitted by air.

### Mask use before the Covid-19 pandemic: a series of health crises

The surgical mask was met with reluctance as it moved from health practices to the public sphere, demonstrating the extension of medical power into individual lives [6]. It was first designed and disseminated for hospital staff in contact with patients with infectious diseases, following the spread of microbiological knowledge in the 1890s [7]. The cloth worn by surgeons over their mouths in the operating room was later worn in the open by Chinese and American physicians during the pneumonic plague pandemics in 1910 and influenza in 1918 [8]. Campaigns to promote masks and other social distance measures in 1918 had already met with resistance [9]. The widespread use of surgical masks was later made possible by the invention of the disposable plastic mask in the 1950s [10]. The SARS (Severe Acute Respiratory Syndrome) epidemic of 2002–2003 made wearing masks commonplace in Asian societies not only in times of health crisis but also in ordinary times, for example as a protection against air pollution [11].

In 2011, a survey in Tokyo of a sample of 120 people showed that half wore masks regularly while the other half rarely or never wore them. About 100 of them said that the mask protected them from chronic communicable diseases. Young women reported wearing the mask to avoid sunburn or simply to hide the fact that they had not had time to apply makeup. Social or occupational compulsion was cited as the major reason for wearing the mask, even though the state had not made it mandatory [12]. In China, mask wearing is seen by individuals as a way to take responsibility and mobilize in the face of an epidemic. It was not mandated by the Chinese state during the SARS crisis, but it externally reinforced orientalizing prejudices describing Chinese citizens as “gagged by power” [13].

Mask stockpiling had been one of the measures by which states, coordinated by the World Health Organization, prepared for future pandemics to limit their health and economic consequences since the SARS crisis [14]. The H1N1 pandemic in 2009 did not lead to widespread mask use. For example, a study in Mexico City showed that masks were very quickly removed when the perception of infectious risk decreased [15].

### Mask use during the Covid-19 pandemic: material and symbolic dimensions

At the beginning of the Covid-19 pandemic, caused by a virus similar to that of SARS, most states recommended the wearing of masks exclusively for health personnel, in a context of global shortage of mask manufacturing and delivery. Citizens therefore had to respond to conflicting messages that masks were more or less useful in the public space depending on their availability at the hospital. In France, for example, the unavailability of masks at the beginning of the pandemic led the government to say they were useless before imposing them when masks were available, blurring the rationalizations associated with its use [16]. The idea that masks were readily available was not immediately accepted at the beginning of the Covid-19 pandemic, due to the combined effect of their scarcity and price. In Senegal, for example, the first masks available to the population were sold at 500 FCFA each (about $1), while the minimum monthly salary is 59,000 FCFA.

Early studies on mask wearing against Covid-19 have highlighted several resistance factors, quite similar to those analyzed in the context of the H1N1 pandemic: difficulty in perceiving the severity of the disease, physical and social discomfort, barriers to communication of information, perception of identity and autonomy, social pressures etc. [17]. A survey conducted in April 2020, for example, showed that 76% of Germans found it “weird” to wear a mask in public spaces but that 80% of them were willing to put the mask on in the street if it was legally required [18]. In Ghana, a survey of truck drivers found that few wore masks in their vehicles, and fewer still wore them properly, but those who did were more likely to cite personal safety and the safety of their loved ones (92%) than social pressure (from the President or their boss) or legal obligation In Saudi Arabia, the requirement to wear masks in public, coupled with the availability of low-cost masks, resulted in a mask-wearing rate of 87% in public spaces and 80% in workplaces, although 32% reported discomfort when using it [19]. A study in Spain has showed that four factors were involved in compliance to regulations regarding mask-wearing: perception of friends and family’s compliance, trust in science, perception of the effectiveness of the mask, perception of risk of infection [20].

Surveys have shown a differential in mask acceptability according to age and gender [21]. Older people wear masks more often, which may be explained by their greater susceptibility to so-called “severe” forms of the disease. In most surveys, women are also more likely to wear masks than men. Women more often emphasize the material discomfort of the mask - for breathing in particular - and men the symbolic discomfort - mainly for communication [22]. In countries with an Islamic culture, women’s veiling may have been a factor in favor of wearing masks because there was no reluctance to hide one’s face in public space [23]. Conversely, in countries marked by opinion movements opposed to the Islamic veil, there was greater resistance to wearing the mask [24, 25].

The mask is indeed a “frontiers object” [26] that defines the person in his or her relationship with others, by separating what can be hidden and what can be shown: *persona* means “mask” in Latin [27]. It is also a material object which can be standardized according to industrial norms or, on the contrary, appropriated by artisanal techniques, which changes its availability and its uses. In this article, we take the material and symbolic use of the mask as an indicator of relations between individuals and the State, in its double function of regulator of public health and provider of protective equipment. We therefore speak of techniques of governing masks, in the sense that these objects orient individuals’ behaviors in a public space where they must arbitrate between authority and intimacy.

### Objective and hypothesis

We want to show that the obligation to wear a mask is not perceived equally according to how the State represents what a person is in the face of a potentially infected environment. In this way, we are interested in the biopolitics of mask wearing or the government of masks, defined as a “conduct of conducts” by which the State regulates individual behaviours [6].

To test this hypothesis, we interviewed people in what we call sentinel territories, in the sense that they are particularly sensitive to the effects of emerging diseases for which the State needs to get prepared [28]. Because of the density and diversity of their populations, but also because of the precariousness of their living conditions, emerging diseases develop there more than elsewhere and are the subject of surveillance programs. Wearing a mask to limit the effects of a pandemic is therefore one of the measures taken to anticipate it. These territories also have in common that they have a large Muslim community. It was therefore possible to test whether religious conceptions of the individual, i.e. appropriate ways of presenting oneself in the public space, could influence public health practices. These territories are therefore good sites to observe and compare the symbolic and material government of masks by the State in the regulation of individual behaviours.

The two sentinel territories selected for this study were: Seine-Saint-Denis, a hyper-urbanized department north of Paris (France) and the city of Dakar, the capital of Senegal. Seine-Saint-Denis was the French department most exposed to the Covid-19 epidemic, with 2500 people dying from the disease in August 2021 for 1.6 million inhabitants, leading to an excess mortality of 130%. This high number is explained in particular by the concentration of the population in small dwellings and by the lack of hospital resources. Immigrant populations, particularly from North Africa, represent 30% of the department’s population and are more exposed to the pathological effects of the virus [29]. In Senegal, as of May 2022, 86,230 cases of Covid-19 had been confirmed, officially resulting in 1,966 deaths. Almost half of the cases (more than 40,000) were in the city of Dakar, which has a population of just over one million. The city of Dakar hosts 23% of the population and half of the country’s hospitals. Monitoring of the pandemic by health authorities seems to have been better there than elsewhere, even if the number of deaths reported for Covid remains very low in Senegal, as in sub-Saharan Africa in general.

## Data and method

### Population sample

This study was conducted from October to November 2021 on a sample of 606 individuals in Dakar and from September 2021 to May 2023 250 individuals in Seine-Saint-Denis, at a time when wearing a mask was mandatory in the public space in France as in Senegal.

As in other studies conducted by our team in Senegal [30], the Dakar sample was constructed using the combined quota method (cross-section by age, gender and town of residence) in order to reach a representative population aged 20 and older living in the department of Dakar. Data from the *Agence Nationale de la Statistique et de la Démographie* from the last census (2013) were used. The quota variables used were gender (male/female), age (20–29 / 30–39 / 40–49 / 50–59 / 60 and over, with an upper age limit of 100 years, but concretely, the oldest participant was aged 90) and town of residence. The towns were grouped by the four arrondissements making up the department of Dakar: Plateau-Gorée (5 towns), Grand Dakar (6 towns), Parcelles Assainies (4 towns) and Almadies (4 towns). Practically, this method requires constructing a sample that reflects the proportions observed in the general population. For example, according to the last census, women aged 20–29 living in town of Medina (arrondissement of Plateau-Gorée) represented 1.5% of the population aged 20 and older living in the department of Dakar. The sample has been constructed so as to reflect this proportion and include 9 women aged 20–29 living in this town.

In order to limit any bias associated with this sampling method, the investigators worked at different moments of the day (and sometimes on Saturday and Sunday) and, in each town of residence, began their investigation from different starting points each day. Eight trained investigators (PhD students in Sociology and Medicine) started out each day from different points in each town to interview individuals in Wolof, Haalpulaar or French in every third home, i.e. the dwelling behind every third front door or entrance gate. Investigators had a certain number of individuals to interview (women aged 20–29 / men aged 20–29 / women aged 30–39 / men aged 30–39 / women aged 40–49 / men aged 40–49 / women aged 50–59 / men aged 50–59 / women aged 60 and over / men aged 60 and over) to meet the quotas. Only one person was selected as a respondent in each home. Investigators went to the house, inquired about the inhabitants and then chose the first person they saw who met the characteristics needed for the quotas.

In Seine-Saint-Denis, middle school students aged 13 to 15 interviewed 250 people in public places (markets, squares) in Blanc-Mesnil (175 people) and Montreuil (75 people) during the day. The majority of respondents were adults (60% between 30 and 60 years old) and the number of women was equal to the number of men (52%). However, the sample was not drawn with reference to a predefined distribution; it is smaller and less representative than the Dakar sample. The sampling techniques were different because we initially built the questionnaire with school students in Seine-Saint-Denis on a rudimentary basis and then decided to use the same questionnaire in Dakar with a more rigorous method. The two samples can therefore only be compared with caution.

However, this comparison is made possible by the fact that the questionnaires used in France and Senegal were exactly the same, and produce interesting and clear results. There were a few minor differences between the two questionnaires: age was assessed with two questions in Dakar in order to obtain the official and, above all, the real age of the individuals; ethnicity was asked in Dakar but not in Seine-Saint-Denis. The questionnaires could be translated in Wolof in Senegal, in Arabic and Tamul in Seine-Saint-Denis. In both territories, data were collected through directed, individual interviews carried out face-to-face using a questionnaire. In-person interviews ranged from 15 min to more than 30 min, depending on respondent availability and desire to talk.

This research has been conducted in accordance with the declaration of Helsinki. Written informed consent was obtained from participants.

### Questions about mask use against Covid-19

Two issues can be distinguished in the relationship between individuals and the State through mask government: scientific authority by contrast with other symbolic forms of representation, and material use in public space.

Several questions aimed to determine whether individuals adhere to public health campaigns recommending mask use and what the basis of their confidence was. The first question measured adherence: Do you think that wearing a mask is effective against Covid-19? Two other questions were used to determine the need for the mask: Do you use the mask to protect yourself? Do you use the mask to protect others? A fourth question asked about trust figures in the health campaign: Who are the appropriate authorities to recommend wearing the mask? Three responses were offered: Scientific authorities (medical doctor, pharmacist, expert in the media); religious authorities (imam, priest, pastor…); political authorities (mayor, deputy, president of the Republic)?

Other questions focused more on the conditions of material use of the mask in a public space. One question concerned the places where wearing a mask is considered necessary: what are the spaces in which you wear a mask? Six answers were proposed, going from the most public to the most private spaces: public transport; market, supermarket, shops; professional office; in the street; hospital; family, home. The effects of wearing a mask were assessed by a question with four response modalities: do you feel uncomfortable wearing the mask? To breathe; to look at others; to talk; for other reasons (please specify). We didn’t include answers such as “I feel embarrassed” or “I feel oppressed”. Finally, a last question concerned the method of obtaining masks, indicating the associated economic cost: how did you obtain your masks? Four answers were possible: purchased disposable; purchased in cloth; donated by an administration; made in cloth.

In Seine-Saint-Denis, when individuals didn’t want to answer questions from school students, they would just pass away. In Dakar, when they thought the researchers were working for the State to control mask wearing, time was taken to explain the purpose of the research (understanding how masks changed daily life) and masks were offered in compensation.

### Analyses

Bivariate analyses (Chi-square tests) were used to compare data collected in France and Senegal. Although the sample design methods were different, the survey populations were similar in age and gender distribution.

## Results

### Adherence to public health campaigns and legitimacy of authorities

The Seine-Saint-Denis sample had a much lower proportion of people who said they believed in the effectiveness of the mask (75%) than in Dakar (89% in Dakar; Chi2(1ddl) = 27.44; p < 0.001). Similarly, Seine-Saint-Denis was distinguished by a lower proportion of people who reported wearing the mask to protect themselves (47% versus 94% in Dakar; Chi2(1ddl) = 248.64; p < 0.001); and a lower proportion of people using the mask to protect others (53% versus 87% in Dakar; Chi2(1ddl) = 116.39; p < 0.001).

In both Dakar and Seine-Saint-Denis, the authorities considered most competent to recommend mask use were scientific authorities, followed by political and religious authorities (Table 1). Despite this general result shared by both populations, large disparities were observed: Dakar residents gave more credence to scientific authorities (87%) than Seine-Saint-Denis residents (58%, Chi2(1ddl) = 66.85; p < 0.001). Similarly, Dakar residents were more likely to consider religious authorities to be in a position to make recommendations about masks than their French counterparts (27% vs. 2%; Chi2(1ddl) = 70.16, p < 0.001). On the other hand, in both populations, less than half of the individuals felt that political authorities were legitimate in recommending wearing masks.

**Table 1:** Which authorities are responsible for recommending the use of masks?

Types of authority	Dakar (N = 606)	Seine-Saint-Denis (N = 250)
Scientific	87%	58%
Political	43%	40%
Religious	27%	2%

### Places where masks are worn and economic conditions

In Dakar and Seine-Saint-Denis, public transport was where the mask was most often worn: 95% and 62% of individuals respectively. This difference is significant (Chi2(1ddl) = 155.02; p < 0.001): the inhabitants of Dakar wear the mask more often in public transport than those of Seine-Saint-Denis. In both territories, the mask is then most often worn in markets or supermarkets: by 81% of individuals in Dakar and 60% in Seine-Saint-Denis (Chi2(1ddl) = 41.59; p < 0.001). While the workplace ranked third in Seine-Saint-Denis, it ranked only fifth in Dakar, after the hospital and outdoor public spaces (Table 2). Finally, the home was only in last place in both populations, but with a significant difference, since while barely 6% of Dakar residents said they wore a mask in their home, 19% did so in Seine-Saint-Denis (Chi2(1ddl) = 34.95; p < 0.001).

**Table 2:** In which spaces do you wear the mask ?

Types of spaces	Dakar (N = 606)	Seine-Saint-Denis (N = 250)
Public transport	95%	62%
Markets	81%	60%
Hospital	48%	41%
Office	34%	53%
Street	45%	47%
Home	6%	19%

In Dakar, the vast majority of masks were disposable, purchased by individuals (81%) and very few were provided by the administration (7%), but a significant proportion of the population created their own masks (9%). In Seine-Saint-Denis, most of the masks were also disposable and purchased by individuals (60%), but more than a quarter of the individuals had received their masks from the state (27.5%). On the other hand, very few (3%) used self-sewn masks.

In Dakar, the most important discomfort associated with wearing a mask was respiratory (69.6%), followed by discomfort in speaking (26.9%) and discomfort in seeing (6.1%). In the Seine-Saint-Denis sample, the discomfort reported was more related to communication conditions: 47% considered the mask to be a hindrance to breathing, 38% to speaking and 8% to seeing.

## Discussion

The main result of our comparative study is that individuals who answered the questionnaire in Seine-Saint-Denis received more masks from the State than those who were interviewed in Dakar, and yet they were more suspicious of scientific authority of the mask efficacy. This can be explained by the fact that the State initially reserved the wearing of masks for hospital staff in a context of shortage of medical facilities, which is reinforced in a densely populated region like Seine-Saint-Denis. The experience of precariousness and vulnerability leads some people to wear the mask in the intimate space, but this form of concern for loved ones remains hidden, while not wearing a mask in the public space was for others a way to challenge the authority of the State. From a symbolic point of view, the mask is for many people an intrusion of the State into the private sphere, which hinders physical contact between family members. On the contrary, from the material point of view, the mask is a need that manifests the presence of the welfare State to watch over the health of the population. To analyze this contradictory dual relationship to the State, we can distinguish the “left hand of the State” that gives masks to individuals and the “right hand” that punishes them if they do not wear masks [31]. However, this defiance towards the State in Seine-Saint-Denis is probably not representative of France as a whole, as the department has particular demographic and socio-economic characteristics.

By contrast, the State has made little commitment to pandemic control measures in Senegal, with only a few curfews. People cope with masks pragmatically in a context where public health is not a priority. Religious authorities then play a more important role in Dakar, as they frame the chains of giving and counter-giving through forms of religious offering [32]. Thus, it is understandable that in our samples individuals more readily refer to religious authorities about whether the mask should be worn in public. In Dakar, scientific, political, and religious authorities appear as more legitimate to promote the wearing of the mask than in Seine-Saint-Denis. Indeed, while the State plays a central role in France in “conducting conduct,“ West Africa is characterized by a multiplicity of legitimate power arrangements and a more flexible form of biopolitics [33]. If individuals are able to use these powers when their injunctions are divergent, in the case of the pandemic, they were unanimous in October 2020, recommending without reservation the wearing of a mask. At the Magal in Touba - the great pilgrimage of the Mourides - the State, religious and medical authorities all urged individuals to wear masks in the public sphere, with a much better result in terms of adherence to public health campaigns than what was observed in France.

Our results thus corroborate classical analyses of governmentality in France and Senegal, that is the relation between the State and the population in times of public health emergency [34]. While the French State has historically relied on a strong scientific expertise imposed from a center to marginal territories, the Senegalese State, since French colonization, had to negociate with a plurality of powers, particularly religious. The notion of sentinel territories that allowed us to compare Dakar and Seine-Saint-Denis, as territories exposed to emerging diseases and requesting interventions of the State, thus take different meanings depending on the history of State power and biopolitics.

The main limitation of this survey concerns the sampling methods used in each of the sites studied. The pandemic made it impossible to carry out an absolutely homogeneous survey and the methods had to be adapted to the particular contexts of this work. Thus, the comparisons made must be considered with caution. Another important limitation of our study is the absence of psychological responses to the question “do you feel uncomfortable wearing the mask? Thus, psychological discomforts were not analyzed in this study. This would have allowed us to broaden the spectrum of factors analyzed in this study.

## Conclusion

This study is the first comparison of mask wearing in two widely separated areas. Its results, which are limited by the comparability of the samples constructed, confirm other studies on the impact of age and gender in mask wearing, showing that older people and women follow mask wearing recommendations more. Above all, it clearly shows contrasting relationships to the State in the public space, and thus different forms of mask government for those who decide to wear them or not, depending on the influence of religious authorities in public life. A State that is both more coercive and more protective, such as France, produces a lower level of adherence to mask-wearing recommendations than a State where prescribing authorities, particularly religious, are more diverse, and where individuals make masks themselves. While Seine-Saint-Denis and the city of Dakar are sentinel territories with regard to the spread of the epidemic, this notion does not have the same meaning in the use of masks: the Paris suburbs were more exposed than other French regions to the ambivalence of the State and its hesitations in times of pandemic, while the Senegalese capital played more the role of an outpost for the sanitary measures followed in Africa, in particular through the supervision of a major pilgrimage.

This study, despite its methodological flaws due to the conditions of social science research during the pandemic, has important implications for the development of effective public health campaigns in sentinel territories, particularly during future airborne epidemics. It suggests that a successful campaign targeting people’s proper behaviors related to protective masks should include religious authorities to exert symbolic power at the same level as scientific and political authorities, and people’s capacities to manage the material conditions of mask making and wearing. This article shows, relying on quantitative data and on theoretical tools from social sciences, that wearing protective masks engages people’s intimacy (their *persona*) in a public space regulated by state interventions in times of emergency.

## Data Availability

The data produced by the investigation in Dakar are available in a file entitled “Base masques 600”. The data produced by the investigation in Seine-Saint-Denis are available on a padlet: https://padlet.com/F93padlet/vbs017w8tysryvef.

## References

[1] Greenhalgh T, Schmid MB, Czypionka T, Bassler D, Gruer L (2020). Face masks for the public during the covid–19 crisis. BMJ.

[2] Lai A, Poon C, Cheung A (2012). Effectiveness of facemasks to reduce exposure hazards for airborne infections among general populations. J Royal Soc Interface.

[3] Cheng VC, Wong SC, Chuang VW, So SY, Chen JH, Sridhar S (2020). The role of community-wide wearing of face mask for control of coronavirus disease 2019 (COVID-19) epidemic due to SARS-CoV-2. J infectiology.

[4] Ike JD, Bayerle H, Logan RA, Parker RM (2020). Face Masks: their history and the values they communicate. J Health Community.

[5] Zhang YSD, Leslie YH, Sharafaddin-Zadeh Y, Noels K, Lou NM (2021). Public health messages about face masks early in the COVID-19 pandemic: perceptions of and impacts on canadians. J Community Health.

[6] Foucault M (1976). « *Il faut défendre la société* ». *Cours au Collège de France*.

[7] Shooter RA, Smith MA, Hunter CJ. A study of surgical masks. British J Surgery 1959 47: 246–49 10.1002/bjs.18004720312. 10.1002/bjs.18004720312 14446158

[8] Lynteris C (2018). Plague Masks: the visual emergence of anti-epidemic Personal Protection Equipment. Med Anthropol.

[9] Tomes N (2010). Destroyer and teacher”: managing the masses during the 1918–1919 influenza pandemic. Public Health Rep.

[10] Strasser B, Schlich T (2020). A history of the medical mask and the rise of throwaway culture. Lancet.

[11] Kleinmann A, Watson J (2006). SARS in China. Prelude to Pandemics.

[12] Burgess A, Horii M (2012). Risk, ritual and health responsabilisation: Japan’s safety blanket of surgical face mask wearing. Sociol Health Illn.

[13] Sin MSY (2016). Masking fears: SARS and the politics of public health in China. Crit Public Health.

[14] Lakoff A (2017). Unprepared. Global Health in a time of emergency.

[15] Condon BJ, Sinha T, Who is that Masked Person. The Use of Face Masks on Mexico City Public Transportation during the Influenza a (H1N1) outbreak (July 4, 2009). Health Policy. 2010 Apr;95(1):50–6. 10.2139/ssrn.1429824. 10.1016/j.healthpol.2009.11.009 19962777

[16] Deroche C, Jomier B, et Vermeillet S. Santé publique: pour un nouveau départ. Leçons de l’épidémie de covid-19. Rapport du Sénat; 2021.

[17] Shelus VS, Frank SC, Lazard AJ, Higgins ICA, Pulido M, Richter APC (2020). Motivations and barriers for the Use of Face Coverings during the COVID-19 pandemic: messaging insights from Focus Groups. Int J Environ Res Public Health.

[18] Rieger MO (2020). To wear or not to wear? Factors influencing wearing face masks in Germany during the COVID-19 pandemic. Social Health Behaviour.

[19] Agyemang E, Agyei-Mensah S, Kyere-Gyeabour E (2021). Face Mask Use among Commercial Drivers during the COVID-19 pandemic in Accra. Ghana Journal of Community Health.

[20] Cabrera-Álvarez P, Hornsey MJ, Lobera J. Determinants of self-reported adherence to COVID-19 regulations in Spain: social norms, trust and risk perception, Health Promotion International 37 (6), December 2022, daac138, 10.1093/heapro/daac138. 10.1093/heapro/daac138 PMC9620366 36300700

[21] Haischer MH, Beilfuss R, Hart MR, Opielinski L, Wrucke D, Zirgaitis G et al. Who is wearing a mask? Gender-, age-, and location-related differences during the COVID-19 pandemic. PLoS ONE 2020 15(10): e0240785. 10.1371/journal.pone.0240785. 10.1371/journal.pone.0240785 PMC7561164 33057375

[22] Howard MC (2021). Gender, face mask perceptions, and face mask wearing: are men being dangerous during the COVID-19 pandemic?. Personal Individual Differences.

[23] Al Naam YA, Elsafi SH, Alkharraz ZS, Alfahad OA, Al-Jubran KM. Al Zahrani EM Community practice of using face masks for the prevention of COVID-19 in Saudi Arabia. PLoS ONE 2021 16(2): e0247313. 10.1371/journal.pone.0247313. 10.1371/journal.pone.0247313 PMC7894919 33606830

[24] Inglis D, Almila AM (2020). Un-masking the Mask: developing the sociology of facial politics in pandemic Times and after. SocietÃ Mutamento Politica.

[25] Chen M (2012). Masked States and the screen between disability and security. Women Stud Q.

[26] Tsang PM, Prost A (2021). Boundaries of solidarity: a meta-ethnography of mask use during past epidemics to inform SARS-CoV-2 suppression. Br Med J Global Health.

[27] Mauss M (1950). Une catégorie de l’esprit humain: la notion de *personne*, celle de “moi”. Sociologie et anthropologie.

[28] Keck F (2020). Avian Reservoirs. Virus hunters and birdwatchers in chinese Sentinel posts.

[29] Brun S, Simon P. L’invisibilité des minorités dans les chiffres du Coronavirus: le détour par la Seine-Saint-Denis. in: Solène Brun et Patrick Simon (dir.), Dossier « Inégalités ethno-raciales et pandémie de coronavirus », *De facto* 2020, 19, http://icmigrations.fr/2020/05/15/defacto-019-05/.

[30] Duboz P, Macia E, Diallo AH, Bergouignan A, Seck SM (2021). The good life in rural and urban Senegal: a qualitative and quantitative study. PLoS ONE.

[31] Bourdieu P (2012). Sur l’État. Cours au Collège de France (1989–1992).

[32] Bondaz J, Bonhomme J (2015). L’Offrande de la mort. Une rumeur au Sénégal.

[33] Macia E, Chevé D, Havard JF (2022). Biopolitiques en Afrique de l’Ouest - Mondes de vie, santé, populations.

[34] Bayart JF (2017). L’Etat en Afrique. La politique du ventre.

